# CT-guided Percutaneous Microwave Ablation Combined with Local Radiotherapy or Chemotherapy of Malignant Pulmonary Tumors

**DOI:** 10.2174/0118744710261655231214105406

**Published:** 2024-01-09

**Authors:** Rongde Xu, Jingjing Chen, Daohua Chen, Xiaobo Zhang, Wei Cui, Yi Deng, Danxiong Sun, Bing Yuan, Jing Li

**Affiliations:** 1 Department of Interventional Radiology, Guangdong Provincial People's Hospital (Guangdong Academy of Medical Sciences), Southern Medical University, Guangzhou, Guangdong, 510080, China;; 2 Department of Pulmonary and Critical Care Medicine, Guangdong Provincial People's Hospital (Guangdong Academy of Medical Sciences), Southern Medical University, Guangzhou, Guangdong, 510080, China;; 3 The Second School of Clinical Medicine, Southern Medical University, Guangzhou, Guangdong, 510080, China;; 4 School of Automation, Guangdong University of Technology, Guangzhou, Guangdong, 510006, China;; 5 Department of Pulmonary and Critical Care Medicine, The First People's Hospital of Yunnan Province. The Affiliated Hospital of Kunming University of Science and Technology, Kunming, Yunnan, 650000, China;; 6 Medical School, Kunming University of Science and Technology, Kunming, Yunnan, 650000, China

**Keywords:** CT, microwave ablation, radiotherapy, chemotherapy, malignant pulmonary tumors, BAI

## Abstract

**Background and Objective:**

The study aimed to investigate the clinical efficacy of CT-guided microwave ablation (MWA) combined with ^125^I seed implantation or bronchial arterial infusion (BAI) chemotherapy in the treatment of malignant pulmonary tumors.

**Methods:**

A total of 56 patients who underwent MWA, MWA combined with ^125^I particle implantation, or MWA combined with BAI chemotherapy for advanced lung cancer or metastatic lung cancer from January 2015 to June 2021 in Guangdong Provincial People’s Hospital were enrolled. Among them, 21 patients were treated with MWA (MWA), 18 with MWA combined with ^125^I seed implantation (MWA+^125^I), and 17 with MWA combined with BAI chemotherapy (MWA+BAI). The short-term outcomes, complications, Eastern Cooperative Oncology Group (ECOG) performance score (Zubrod-ECOG-WHO, ZPS), survival, and factors related to survival were compared between the three groups.

**Results:**

The response rate of the MWA group (9.52%) was significantly lower than that of the MWA+^125^I group (50.00%) and MWA+BAI chemotherapy group (47.06%), and the differences were statistically significant (*p* < 0.05). The incidence of complications in the MWA, MWA+^125^I, and MWA+BAI chemotherapy groups was 47.62%, 55.56%, and 52.94%, respectively, with no significant difference (*p* > 0.05). Three months after the treatment, the ZPS of the MWA+^125^I and MWA+BAI chemotherapy groups was significantly lower than before treatment and significantly lower than that of the MWA group in the same period; the differences were statistically significant (*p* < 0.05). The median survival time of the MWA+^125^I group was 18 (9.983, 26.017) months and that of the MWA+BAI chemotherapy group was 21 (0.465, 41.535) months, both of which were higher than that of the MWA group [11 (6.686, 15.314) months]; the differences were statistically significant (*p* < 0.05). Cox regression analysis was performed on the factors related to survival and revealed treatment mode as a protective factor [HR = 0.433, 95% CI = (0.191, 0.984), *p* = 0.046]. Other factors, such as gender, age, and tumor size, did not independently affect survival.

**Conclusion:**

CT-guided MWA combined with ^125^I seed implantation and MWA combined with BAI chemotherapy are safe and effective for the treatment of advanced lung cancer and metastatic lung cancer, and can control tumor progression and prolong survival time.

## 1 INTRODUCTION

Globally, lung cancer is the second most common malignancy with the highest death rate [1], while in China, it ranks first in both incidence and mortality [2]. Statistics from 2020 show that the incidence and mortality rate of lung cancer in China are higher than the global average [2]. According to American epidemiological data, more than 57% of lung cancer patients have distant metastases at the time of diagnosis [3]. According to Chinese data, stage III-IV lung cancer accounts for 64.6% of all lung cancers [4]. As a result, the majority of patients have already lost the opportunity to undergo surgery by the time of their initial diagnosis. In addition, the lungs are the second most common location of metastases, and more than one-third of patients suffering from malignant solid tumors develop pulmonary metastases [5].

As cancer treatment advances, a major trend in lung cancer treatment comprises precise, minimally invasive, or non-invasive local approaches. These include video-assisted thoracoscopic surgery (VATS) and stereotactic body radiation therapy (SBRT). However, such approaches also present limitations [6]. Although VAST is still a preferred method, it is not suitable for patients who refuse surgery, have recurrent or metastatic lesions, or have underlying diseases that are not suitable for surgery. Regarding SBRT, the size and location of the tumor can affect its therapeutic effect. Therefore, thermal ablation is an emerging approach for the treatment of primary and metastatic lung malignancies. The three major modalities of ablation are radiofrequency ablation (RFA), microwave ablation (MWA), and cryoablation. Each of these has its own advantages and disadvantages. The advantage of MWA compared to the other two techniques is its capacity to treat large lung tumors faster, with a reduced perivascular heat sink effect because of the structure of the lung [7]. The clinical use of MWA to treat lung cancer is now widespread [8]. When MWA is used to treat stage IA (the new 8th edition of TNM [9]) non-small cell lung cancer (NSCLC), a curative outcome is achieved in the majority of cases [10, 11]. For early-stage lung cancer, previous randomized controlled trials have demonstrated no significant differences in survival rates between thermal ablation and surgical resection [12]. Alternatively, thermal ablation can be used as palliative therapy for advanced lung cancer to reduce the tumor burden [13]. In metastatic lung cancers, such as pulmonary metastases from colon cancer, thermal ablation has shown excellent local control rates (62-91%) [14]. Control rates achieved in the lung with thermal ablation rival those that can be obtained with SBRT [15]. Tumor size, tumor localization, and vicinity to vital organs are determinants of local tumor control with MWA. Therefore, for lung tumors larger than 5 cm or lesions with special locations, Chinese guidelines [16] suggest that thermal ablation be used in combination with other treatments. Currently, MWA combined with iodine-125 (^125^I) seed implantation and MWA combined with bronchial arterial infusion (BAI) chemotherapy are widely used. ^125^I has a persistent local radioactive effect on tumors that inhibits the growth of tumor cells and causes degeneration and necrosis of the tumor tissues [17]. Studies have proven that ^125^I can be used as a complementary therapy for residual lesions in patients with hepatocellular carcinoma (HCC) after RFA to prolong progression-free survival [18]. In recent decades, BAI chemotherapy has been proven to be a safe, efficacious, and economical approach for treating advanced lung cancer and metastatic lung cancer [19].

There are a few clinical reports on the combination of MWA and ^125^I seed implantation or BAI chemotherapy in the treatment of advanced lung cancer or metastatic lung tumors. This study aimed to investigate the clinical efficacy and safety of MWA alone, MWA combined with ^125^I seed implantation, and MWA combined with BAI chemotherapy in advanced lung cancer and metastatic lung tumors.

## 2 MATERIALS AND METHODS

### 2.1 Study Population

**Fig. (1):**
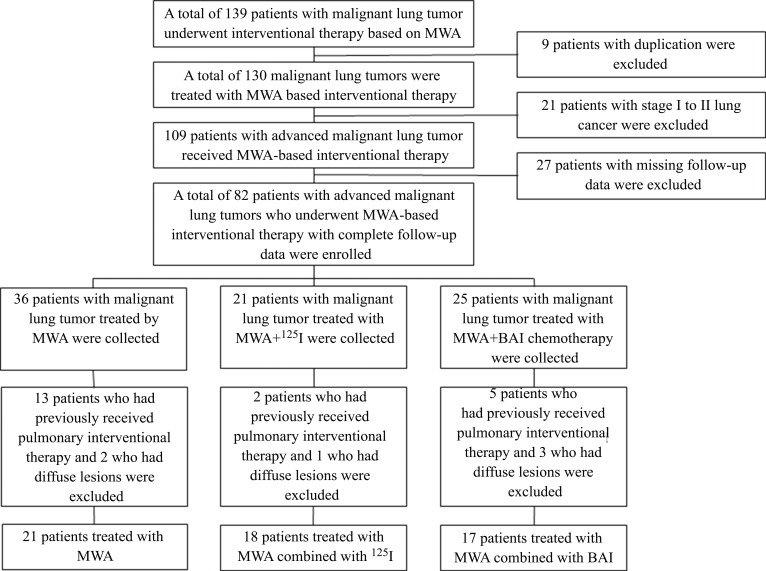
A flow chart showing study group enrollment.

Patients with advanced lung cancer or metastatic lung tumors who received MWA or MWA combined with ^125^I seed implantation or MWA combined with BAI chemotherapy in Guangdong Provincial People’s Hospital from January 2015 to June 2021 were retrospectively sought out. A total of 139 patients with malignant lung tumors who underwent MWA-based interventional therapy were included, and a total of 83 patients were excluded according to the inclusion and exclusion criteria (Fig. **1**). A total of 56 patients were enrolled, including 21 patients treated with MWA (MWA), 18 patients treated with MWA combined with ^125^I seed implantation (MWA+^125^I), and 17 patients treated with MWA combined with BAI chemotherapy (MWA+BAI). Ethical clearance was obtained from the Ethics Committee of Guangdong Provincial People’s Hospital (ethics approval number KY-Q-2021-133-01). As this study was retrospective and based mainly on medical records, there was no need to obtain signed informed consent forms from the patients and their families again.

### 2.2 Inclusion and Exclusion Criteria

#### 2.2.1 Inclusion Criteria

All the patients were pathologically confirmed to have primary lung cancer or metastatic pulmonary malignancy. Patients enrolled with primary lung cancer were at stages III-IV (the new 8th edition of TNM [9]). All patients were treated with lung tumor ablation for the first time without pulmonary surgery. Patients or family members signed informed consent forms for interventional therapy.

#### 2.2.2 Exclusion Criteria

Cases with missing follow-up information were excluded, as were patients who had diffuse lung metastases and whose tumor size could not be measured, patients with severe organ dysfunction, such as heart failure or other organ failures, patients with coagulation dysfunction or platelets < 50 × 109/L, and those with an Eastern Cooperative Oncology Group (ECOG) [20] physical status score > 2.

## 3 TREATMENT

MWA can be used to treat tumors with small diameters located outside the lung. For large tumors where the therapeutic effect of MWA is poor, MWA can be combined with ^125^I seed implantation. MWA combined with BAI can be used for large tumors or those located near vital organs.

### 3.1 Treatment Method

#### 3.1.1 MWA

All patients underwent plain and contrast-enhanced chest CT within 1 week before surgery to measure the tumor size, location, and relationship to adjacent organs, as well as to carry out other general cardiopulmonary imaging. Routine blood tests, coagulation function tests, liver and kidney function tests, and electrocardiogram (ECG) were performed within 3 days before the operation. Echocardiography was performed in patients over 60 years old. After obtaining the above-mentioned data, a multi-disciplinary consultation with experts in respiratory and critical diseases, thoracic surgery, oncology, and interventional medicine, was conducted. The patients were evaluated before the operation and the ablation plan was formulated. Patients and their families signed informed consent for interventional therapy. Patients were made to fast for more than 4 hours before the operation. Multifunctional ECG monitoring was used to monitor vital signs during the operation. The tumor location and puncture route were determined by 256-slice CT (Philips Healthcare, Amsterdam, the Netherlands) scanning and 3D reconstruction. The operator strictly followed the aseptic operation. Local anesthesia was administered with 2% lidocaine. According to the predicted puncture route, when the MWA needle was inserted near the lesion, another CT scan was performed to determine the distance between the tip and the tumor center. The operator further adjusted the puncture needle to make the tip reach the tumor center. Then, the operator fixed the puncture needle and connected the microwave instrument (MTI-5AT, Nanjing Great Wall Medical Equipment Co., Ltd) and the water circulation cooling instrument. Ablation parameters and time were set according to specific conditions. Following the guidelines strictly, single-point, single-multipoint, or multiple-multipoint MWA was performed according to the size of the tumor. After the operation, a CT scan was performed to evaluate the results of ablation and determine whether there were complications, such as pneumothorax and hemorrhage.

#### 3.1.2 MWA+^125^I

The MWA procedure was the same as described above. Within 1 week of MWA, the number and distribution of ^125^I implants were determined by the treatment planning system. Under the guidance of a CT scan, the 18G seed implantation puncture needle was passed through the tumor center with the tip 5 mm from the tumor edge. After withdrawing the needle core and confirming that no vessels were damaged, ^125^I seeds with 0.5 mCi to 0.8 mCi activity were implanted with an interval of 0.5-1 cm to cover as much of the tumor as possible. The particles should be more than 1 cm away from the heart, main airways, and large vessels to avoid damage to vital structures. A postoperative CT scan was used to evaluate the implantation and complications.

#### 3.1.3 MWA+BAI

Percutaneous femoral artery puncture was performed using the Seldinger method under the guidance of digital subtraction angiography (DSA; Innova 4100-IQ, GE). After arterial ducts, to target the bronchial artery, iodixanol angiography was performed to identify the supplying artery of the tumor and its relationship with the anterior spinal artery. The 2.7F microcatheter was used to superselect into the target artery of the tumor. Gemcitabine 600 mg/m^2^ or pirarubicin 25 mg/m^2^ combined with lobaplatin 30 mg/m^2^ were formulated in 150 mL sterile 0.9% saline or 5% glucose solution. All drugs were infused *via* the catheter into the target artery at a rate of 10 ml/min. The patients underwent CT-guided MWA 1 week after the first round of BAI chemotherapy.

All the invasive operations were performed by a senior doctor and guided by a chief physician.

## 4 OBSERVATION INDICATORS

### 4.1 The Therapeutic Effect in the Three Groups

The efficacy evaluation was based on the grading efficacy evaluation criteria of tumor lesions formulated by the WHO [21] and the CT evaluation of ablation efficacy with reference to “Clinical practice guidelines: image-guided thermal ablation of primary and metastatic lung tumors (2021)” [16], as well as the comprehensive evaluation of the patient’s condition. CT scan findings 3-6 months postoperatively were obtained. Complete response (CR) was indicated by one of the following: i) disappearance of the tumor body; ii) complete cavitation; iii) fibrosis or fibrous scars; iv) tumor shrinkage ≥ 70%; v) presence of solid nodules, but CT enhancement did not suggest contrast enhancement, or no tumor metabolic activity was observed on PET-CT; and vi) presence of atelectasis, but there was no CT contrast enhancement in the lesion or tumor metabolizing activity on PET-CT. Partial response (PR) was indicated by one of the following manifestations: i) 30%-70% tumor shrinkage on CT scan and ii) partial fibrosis of tumor lesions, or necrosis or fluid cyst formation in the center of the lesions. Stable disease (SD) was indicated by lesion reduction < 30% or no cavitation or fluid cystic cavity and no new lesions. Progressive disease (PD) was indicated by the appearance of new lesions or an increase in the size of the existing lesions of ≥ 20%. Response rate (RR) = (CR + PR)/total cases × 100% and disease control rate (DCR) = (CR + PR + SD)/total cases × 100%.

### 4.2 Treatment Safety

The complications occurring in the three groups were recorded, and the incidence of complications in the three groups was compared to evaluate the safety of the three recorded.

### 4.3 Follow-up and Survival Analysis

After discharge, the patients were followed up and analyzed post-operatively. If the patient was followed up in the local hospital, we arranged for a telephonic follow-up. Follow-up parameters included ECOG as standard performance status (PS) and patient survival time. The survival time was measured from the day post-operation to death or the end of follow-up. The follow-up period ended on December 31, 2021. The survival time of the three groups was compared.

### 4.4 Statistical Methods

Data were analyzed using SPSS22.0 statistical software. Measured data have been expressed as mean ± standard deviation, and the data of the three groups were compared using one-way ANOVA. Repeated measures ANOVA was used for data with multiple repeated measures at different time points for the same observation. The chi-squared test was used to compare count data. The Wilcoxon rank-sum test was used for the analysis of rank data. Survival analysis was performed using the Kaplan-Meier method, the log-rank test was used for comparisons, and the least significant difference (LSD) was used for pairwise multiple comparisons. Univariate Cox regression and multivariate Cox regression (method: LR) were used to identify the independent prognostic factors of lung malignancies. With α = 0.05 as the level, *p* < 0.05 was considered statistically significant. Pairwise comparisons of the chi-squared and rank sum tests were made to adjust the testing level α´ = α. The number of comparisons, *p* < α´ has been considered statistically significant for difference.

## 5 RESULTS

### 5.1 Basic Data

The clinical characteristics and basic data of the patients are summarized in Table **1**. A total of 56 patients were included. A total of 21 patients, including 17 males and 4 females, with a mean age of 62.76 ± 11.39 years were included in the MWA group, which included 14 primary lung cancers (four adenocarcinomas, seven lung squamous cell carcinomas, one adenosquamous carcinoma, and two undifferentiated carcinomas) and seven metastatic lung malignancies (two liver cancers, two colon cancers, one gastric cancer, and two urologic malignancies). One case of EGFR (+) and three cases of ALK (+) NSCLC were present. A total of 18 patients, including 13 males and 5 females, with a mean age of 58.39 ± 9.03 years were included in the MWA+^125^I group. The group comprised 7 primary lung cancers (two lung adenocarcinomas, three lymphatic epithelioma carcinomas, and two undifferentiated carcinomas) and 11 metastatic lung malignancies (three liver cancers, two mediastinal malignancies, one prostate cancer, one esophageal cancer, one uterine cancer, one fibrosarcoma, one squamous cell carcinoma of the oral floor, and one gastric cancer). One case of EGFR (+) NSCLC was present. A total of 17 patients were included in the MWA+BAI chemotherapy group, including 13 males and 4 females with a mean age of 58.88 ± 10.67 years. The group included 12 primary lung cancers (seven lung adenocarcinomas, two squamous cell carcinomas, one adenosquamous carcinoma, and two undifferentiated carcinomas) and 5 metastatic lung malignancies (two liver cancers, one colon cancer, one submandibular gland malignancy, and one mediastinal malignancy). There were 2 cases of EGFR (+) and 2 cases of ALK (+) NSCLC. Patients with the gene mutation had undergone molecular targeted therapy, which had failed. In the MWA group, 8 patients (38.10%) had tumors located in the inner two-thirds of the lung field. In the MWA+^125^I group, 14 cases (78.78%) had tumors located in the inner two-thirds of the lung field. In the MWA+BAI chemotherapy group, 13 cases (76.47%) had tumors located in the inner two-thirds of the lung field. Using the chi-squared test, χ^2^ = 8.545 and *p* = 0.014 indicated the distribution of tumor location in the three groups as statistically significantly different. Fisher’s exact probability method was used for further comparison. The proportion of tumors located in the inner two-third band of the lung field in the MWA+^125^I group was significantly higher than that in the MWA group (*p* = 0.014), and the proportion of tumors located in the inner two-third band of the lung field in the MWA+BAI chemotherapy group was significantly higher than that in the MWA group (*p* = 0.020). The adjusted level of α was α´ = 0.025, and the difference was statistically significant (*p* < 0.025). There was no significant difference in other baseline clinical data between the three groups (*p*>0.05).

### 5.2 Comparison of Recent Outcomes

The three groups of patients were re-examined with chest CT scan and contrast-enhanced or PET-CT within 3-6 months of operation. According to the results of imaging and the actual condition of the patients, a comprehensive assessment was performed. The patients were assigned four grades: CR, PR, SD, and PD. Regarding statistical analysis of the data, the rank-sum test was used for grade data, and four grades were assigned, namely CR = 4, PR = 3, SD = 2, and PD = 1. As shown in Table **2**, the mean rank of patients in the MWA group was 19.81, that of the MWA+^125^I group was 33.97, and that of the MWA+BAI chemotherapy group was 33.44 (χ^2^ = 10.422, *p* = 0.005). This may indicate that the patients in the three groups did not experience the same short-term curative effect, and the difference was statistically significant (*p* < 0.05). The Wilcoxon rank-sum test was used for pairwise comparisons of the short-term efficacy of the three groups, and the α level was adjusted to α´ = 0.0167. The mean rank of the MWA+^125^I group was significantly higher than that of the MWA group, indicating that its short-term efficacy was better than that of the MWA group (Z = −2.959, *p* = 0.004), and the difference was statistically significant (*p* < 0.0167). The mean rank of the MWA+BAI chemotherapy group was significantly higher than that of the MWA group, indicating that its short-term efficacy was better than that of the MWA group (Z = −2.583, *p* = 0.014), and the difference was statistically significant (*p* < 0.0167). There was no significant difference between the mean rank of the MWA+^125^I group and that of the MWA+BAI chemotherapy group (*p* > 0.0167).

Fisher’s exact method was used to compare the RR and DCR of the three groups. The test level α was adjusted to α´ = 0.0167. As shown in Table **3**, the RR of the MWA+^125^I and MWA+BAI chemotherapy groups was statistically significantly higher than that of the MWA group (*p* < 0.0167). The DCR of the MWA+^125^I group was statistically significantly higher than that of the MWA group (*p* < 0.0167). There was no significant difference in the RR and DCR of the MWA+^125^I and MWA+BAI chemotherapy groups (*p* > 0.0167).

### 5.3 Safety of Treatment in the Three Groups

The complications of the three groups are summarized in Table **4**. There was no statistically significant difference in the overall incidence of complications among the three groups (*p* > 0.05). There was no statistically significant difference in the incidence of various complications among the three groups of patients (*p* > 0.05). Among the patients with pneumothorax complications in the MWA and MWA+^125^I groups, one needed to be treated with air extraction, while the others had a small amount of pneumothorax that could be absorbed by itself after oxygen therapy and bed rest. Among the patients with pleural effusion in the MWA group, one patient needed pleural effusion drainage treatment, while the others all had a small amount of pleural effusion. After symptomatic support treatment, pleural effusion could be slowly absorbed. All three groups of patients with postoperative chest pain were relieved after treatment with analgesics. Postoperative myelosuppression occurred in one case in the MWA group, one case in the MWA+^125^I group, and two cases in the MWA+BAI chemotherapy group. One week later, a routine blood examination showed that the white blood cell levels were all above 3.5 × 10^9^/L. One case in the MWA+^125^I group and two cases in the MWA+BAI chemotherapy group had mild-to-moderate impairment of renal function. Renal function was re-examined 1 week after treatment to protect the kidney and showed that the creatinine level had returned to normal. There were no serious complications affecting prognosis in the three groups.

### 5.4 ZPS of Three Groups Before and After the Treatment

ECOG developed a simplified activity status rating scale, which became the ECOG scoring standard for physical status (Zubrod-EcoG-WHO, ZPS) [22]. Patients’ activity status was rated on a scale of 0 to 5, with 0 indicating complete normalcy and 5 indicating death [23]. The results in Table **5** show significant differences in the data at each time point (*p* < 0.05). There was no significant difference between the treatment groups (*p* > 0.05). There was also no cross-effect between time and components, indicating that the effect of time was not different between treatment groups, and the difference was not statistically significant (*p* > 0.05). In the first month after the operation, there was no significant difference in ZPS between the three groups compared to the preoperative status (*p* > 0.05). In the third month after the operation, i) there was no significant difference in the ZPS of the MWA group compared to that before operation and in the first month after operation (*p* > 0.05) and ii) the ZPS of the MWA+^125^I and MWA+BAI chemotherapy groups was significantly lower than that before operation and in the first month after operation *(*P < 0.05). In the sixth month after the operation, i) the ZPS in the MWA group was significantly higher than that before the operation and in the first month after the operation (*p* < 0.05); ii) the ZPS in the MWA+^125^I group was significantly lower than that before operation and in the first month after operation, but significantly higher than that in the third month after the operation (*p* < 0.05); and iii) the ZPS in the MWA+BAI chemotherapy group was significantly higher than that before operation and in the first and third months after the operation (*p* < 0.05). Before the operation, there was no significant difference in ZPS between the three groups (*p* > 0.05). In the third month after the operation, the ZPS of the MWA+^125^I and MWA+BAI chemotherapy groups was significantly lower than that of the MWA group (*p* < 0.05). As shown in Fig. (**2**), the ZPS curves of the MWA+^125^I and MWA+BAI chemotherapy groups were below that of the MWA group, which also proved that after treatment, the ZPS of the MWA+^125^I and MWA+BAI chemotherapy groups decreased, indicating that the physical performance scores were better than those of the MWA group.

### 5.5 Survival Analysis of Patients in the Three Groups

**Fig. (2):**
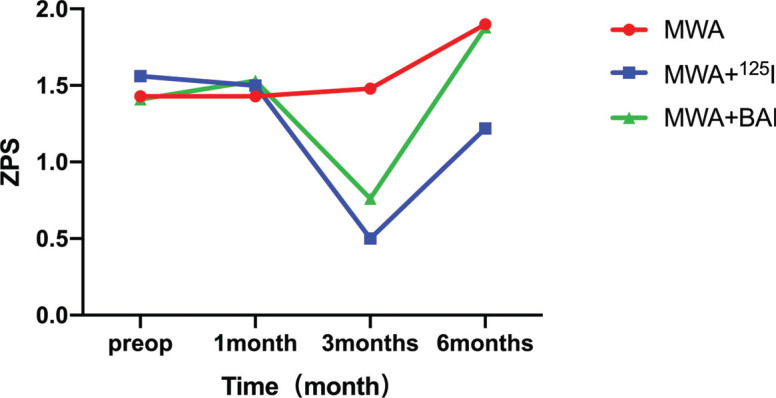
ZPS of three groups before and after treatment.

The 6-month, 1-year, and 2-year survival rates of patients in the MWA group were 90.48%, 42.86%, and 4.76%, respectively, and the median survival time was 11.00 (6.686, 15.314) months. The 6-month, 1-year, and 2-year survival rates of the MWA+^125^I group were 88.89%, 61.11%, and 22.22%, respectively, and the median survival time was 18.00 (9.983, 26.017) months. The 6-month, 1-year, and 2-year survival rates of the MWA+BAI chemotherapy group were 70.59%, 58.82%, and 47.06%, respectively, and the median survival time was 21.00 (0.465, 41.535) months. The Kaplan-Meier and log-rank test methods were used for analysis and graphing (Fig. **2**; χ2 = 6.431, *p* = 0.0401), and there was a statistically significant difference in survival between the three groups (*p* < 0.05). The survival time of the MWA group was significantly lower than that of the MWA+^125^I group (χ2 = 4.787, *p* = 0.029) and MWA+BAI group (χ2 = 3.796, *p* = 0.046), and the difference was statistically significant (*p* < 0.05). There was no significant difference in survival between the MWA+^125^I group and the MWA+BAI chemotherapy group (χ2 = 0.015, *p* = 0.902). As can be seen in Fig. (**3**), the curves of the MWA+^125^I and MWA+BAI chemotherapy groups were above those of the MWA group, indicating that in terms of survival, the effects of MWA+^125^I and MWA+BAI chemotherapy were superior to those of MWA.

### 5.6 Univariate Cox Regression and Multivariate Cox Regression

**Fig. (4):**
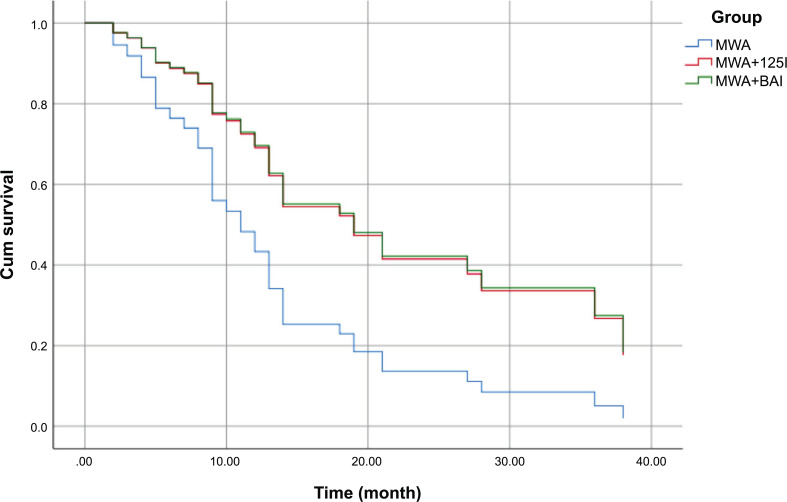
Cox regression survival curve.

Age, preoperative maximum diameter of tumor, and preoperative ZPS were assigned as measured values. The following parameters were taken into account: gender (assignment: female = 0, male = 1), smoking (assignment: no = 0, yes = 1), lung cancer types (assignment: primary lung cancer = 0, metastatic lung cancer = 1), treatment method (assignment: MWA = 1, MWA+^125^I = 2, MWA+BAI chemotherapy = 3), multiple lesions (assignment: single lesion = 0, multiple lesions = 1), and tumor location (assignment: outer one-third zone = 1, inner two-third zone = 2). The results (Table **6**) showed that the final variable that entered the model was the treatment method, with a regression coefficient of 0.433 and a 95% confidence interval of 0.191–0.984, indicating it to be a protective factor. The combined treatment was beneficial for prolonging the survival time, and the difference was statistically significant (*p* < 0.05). The Cox regression survival curve (Fig. **4**) showed that the survival curves of the MWA+^125^I and MWA+BAI chemotherapy groups basically overlapped and were above the survival curves of the MWA group, indicating that MWA+^125^I and MWA+BAI chemotherapy had better effects on survival than MWA. The difference was statistically significant (*p* < 0.05).

## 6 TYPICAL CASES

1. MWA: A 59-year-old male patient was diagnosed with lung adenocarcinoma with brain metastasis in 2016 and had received chemotherapy, targeted therapy, and craniocerebral tumor resection. On June 22, 2017, the patient was treated with MWA after chest CT examination. Chest CT examination 3 months after the operation showed that the right middle lung lesion did not shrink significantly. Figs. (**5A**-**D**) demonstrates the details of the case.
2. MWA+^125^I: A 65-year-old male patient was diagnosed with NSCLC in 2020 and underwent chemotherapy. On January 7, 2021, the right upper lung tumor was treated with MWA. On January 14, 2021, 80 ^125^I seeds were implanted into the right upper lung tumor. Three months later, lung CT reexamination revealed slight shrinkage of the right upper lung cancer lesion. Figs. (**6A**-**D**) depicts the details of the case.
3. MWA+BAI: A 66-year-old male patient was diagnosed with squamous cell carcinoma of the left lung in 2019 and received chemotherapy. On March 25, 2020, a chest CT examination was performed. Lung CT showed total consolidation of the left lung with atelectasis. After examination, the patient received BAI chemotherapy. One week later, the patient was treated with MWA. Three months after the operation, the pulmonary CT showed that the consolidation shadow of the left lung basically disappeared, and scattered nodules and strip shadows could be seen. Figs. (**7A**-**D**) demonstrates the details.

## 7 DISCUSSION

With the advancement of oncology treatment modalities, local thermal ablation, especially image-guided thermal ablation, has been widely used for lung tumors [24], not only in the treatment of early lung cancer [25], but also in the treatment of lung nodules [26]. At the same time, it has been used for the palliative treatment of advanced lung cancer and metastatic lung cancer [25, 27], which could not only improve the quality of life of lung cancer patients but also prolong their survival [28]. MWA makes the polar molecules in the tumor tissue vibrate at a high speed under the action of the electromagnetic field. Through the collision and friction of the molecules, a high temperature of 60-150°C is generated in a short time, thereby causing tumor cell necrosis [29, 30]. Due to the particularity of the lung structure [31], MWA has the advantages of high convection, high thermal efficiency, rapid heating, low thermal precipitation, short ablation time, and complete ablation [26], so it is widely used in the thermal ablation of lung tumors. The guidance modalities for MWA in the treatment of lung tumors include CT, MR, B-ultrasound [30], and the gradually maturing bronchoscopy combined with electromagnetic navigation guidance [32]. CT is still the most commonly used imaging guidance technique. In recent years, many researchers have studied the efficacy and safety of MWA in lung tumors. In a study on 51 MWA-treated patients with perihilar type solitary ground glass nodules less than 3 cm in diameter in the lung, the survival rates without local recurrence and overall survival at 3 years were 98% and 96%, respectively, with a median survival time of 27 months and no MWA-related deaths [33]. At present, most of the studies on MWA alone for the treatment of lung cancer consider single subsolid nodules or stage I lung cancer. For patients with large tumor diameters or special tumor locations, MWA may present defects with incomplete ablation, so a combination of other treatment methods is required. Currently, many combined methods are used, such as MWA with radioactive particle implantation, MWA with local chemotherapy, and MWA with targeted drugs or immunotherapy. ^125^I seeds can activate the p38MAPK/MDM2/p53 signaling pathway and promote the apoptosis of NSCLC cells [34]. The application of BAI chemotherapy in lung tumors has been relatively mature. BAI chemotherapy and drug-eluting embolic (DEE) microspheres have induced overall response rates of as high as 78.3% in stage III/IV lung cancer cases [35]. However, there are a few reports comparing the efficacy of MWA, MWA combined with ^125^I seed implantation local radiotherapy, and MWA combined with BAI chemotherapy. In this study, we have conducted a retrospective analysis of the clinical data of advanced lung cancer and metastatic lung cancer patients who underwent MWA, MWA combined with ^125^I particle implantation, or MWA combined with BAI chemotherapy at the Department of Cancer Intervention of Guangdong Provincial People’s Hospital.

The guidelines [16] recommend combined therapy for patients with a tumor diameter of more than 5 cm or a special tumor location, such as adjacent to the heart, large blood vessels, or main airway, or even invading spinal nerves to address incomplete ablation. It has also been reported that the complete ablation rate of patients with tumors located in the inner two-thirds of the lung field is significantly lower than that of tumors located in the outer one-third [36]. In this study, we found that the tumors of patients in the MWA group were mostly located in the outer one-third of the lung field, while those of patients in the MWA+^125^I group and MWA+BAI chemotherapy group were mostly located in the inner two-thirds of the lung field; the differences were statistically significant (*p* < 0.025). This illustrates that tumors located in the inner two-third bands of the lung field are relatively difficult to ablate completely because of their proximity to the heart, large blood vessels, and large airways, and ablation needs to be combined with other modalities, such as local radiotherapy and chemotherapy, to improve tumor control. This therapeutic principle is consistent with guidelines and other reports in the literature.

The patients enrolled in this study underwent imaging examinations 3-6 months after treatment, and the results were comprehensively evaluated in combination with their disease. Rank statistics was calculated according to the four grades of CR, PR, SD, and PD. MWA combined with ^125^I seed implantation and MWA combined with BAI chemotherapy had better short-term effects than MWA alone, with statistically significant differences (*p* < 0.0167). There was no statistically significant difference between the two combined treatments (*p* > 0.05). The effective rate of MWA combined with ^125^I seed implantation was 50%, and that of MWA combined with BAI chemotherapy was 47.06%, significantly higher than that of MWA alone (9.52%); the differences were statistically significant (*p* < 0.0167). MWA combined with ^125^I seed implantation had an 83.33% control rate, which was also significantly higher than the 42.86% control rate of MWA alone (*p* < 0.0167). The control rate of MWA combined with BAI chemotherapy was 76.47%. The value was not statistically significantly different from that of MWA alone (*p* > 0.0167), but from a clinical perspective, it was higher than that of the MWA-only group. Studies have shown that MWA combined with ^125^I seeds is effective in the local prevention and treatment of retroperitoneal liposarcoma, but the long-term efficacy and survival benefits still need to be comprehensively evaluated [37]. Animal experiments have shown that after arterial infusion or chemoembolization, combined with thermal ablation, liver cancer could be better controlled. In malignant biliary tract tumors, stent implantation combined with intraluminal RFA and hepatic arterial infusion chemotherapy has been reported to be safe and effective [38]. In the treatment of NSCLC, local chemotherapy *via* the bronchial artery with chemotherapeutic microspheres is a feasible and well-tolerated treatment [39]. The results of the present study showed that MWA combined with local radiotherapy or chemotherapy could improve treatment efficacy, which was largely consistent with the results of other studies.

In this study, the incidence of complications in the MWA, MWA+^125^I, and MWA+BAI chemotherapy groups was 47.62%, 55.56%, and 52.94%, respectively. The results showed no significant increase in the incidence of complications in the combined groups (*p* > 0.05). In patients with NSCLC treated with CT-guided MWA, complications occurred in approximately 34.6–57.9% [40] of cases, which was also largely consistent with our results. Pneumothorax is the most common complication after lung ablation, with an incidence of 10% to 60%, with 3.5–40% requiring thoracic drainage [41]. In this study, the incidence of pneumothorax was 9.52%, 16.67%, and 11.76% in patients treated with MWA, MWA + ^125^I, and MWA + BAI chemotherapy, respectively, which was similar to the results of other studies. Moreover, the incidence of pneumothorax did not increase in the two combined treatment groups, and the difference was not statistically significant (*p* > 0.05). In radiotherapy and chemotherapy, the complications of greatest concern are myelosuppression and impairment of liver and kidney function. In this study, no patients with impaired liver function were identified. Myelosuppression occurred in one case (4.55%) in the MWA group, one case (5.88%) in the MWA+^125^I group, and two cases (11.76%) in the MWA+BAI chemotherapy group, and there were no significant differences between the three groups (*p* > 0.05). According to a study from the First Affiliated Hospital of Guangzhou Medical University [42], the incidence of adverse reactions, such as vomiting and myelosuppression due to BAI chemotherapy, was about 6.67%, which was significantly lower than the 22.92% incidence of adverse reactions in patients treated with systemic chemotherapy. Myelosuppression and impaired liver and kidney function have also rarely been reported with local radiotherapy along with ^125^I particle implantation [43]. Regarding the incidence of other complications, such as chest pain, hemoptysis, and pulmonary infection, there was no significant difference found between the combined treatments and MWA alone (*p* > 0.05). Thus, MWA combined with local radiotherapy or chemotherapy in the treatment of lung malignancies did not increase the occurrence of adverse effects compared to MWA alone.

For oncology patients, after undergoing anti-tumor treatment, the recovery of physical function is extremely important. ZPS is primarily evaluated from patients’ symptoms, physical strength, and other dimensions using a scale of 0–5, with lower scores indicating better physical function [44]. In this study, repeated measures ANOVA was used to analyze the ZPS of patients in the three groups before treatment and 1 month, 3 months, and 6 months after the treatment. ZPS changed significantly with time as the main factor (*p* < 0.05). No significant changes in ZPS were observed in the three groups at 1 month after surgery (*p* > 0.05). The reason for this may be related to the adverse effects after ablation, local chemotherapy, and local radiotherapy. Within 3 months of ablation, the destruction of microcirculation and necrosis of tumor tissue and the resulting inflammatory response may cause discomfort, and more than 30% of patients may develop post-ablation syndrome [16]. These reactions can lead to more symptoms in patients and poor physical function evaluation. Using ^125^I implantation to treat patients with advanced lung cancer and airway stenosis, it was found that physical function evaluation improved at least 1 month after the surgery [43]. In the third month after the operation, ZPS was found to be significantly decreased in the two combined groups, and it was significantly lower than that in the MWA group at the same time (*p* < 0.05). This proved MWA combined with ^125^I seed implantation and MWA combined with BAI chemotherapy to be more effective in the recovery of physical function in patients with lung cancer. The recovery of body function has been positively correlated with the therapeutic effect on the tumor [45]. In the 6-month postoperative evaluation, ZPS was found to be significantly increased in the three groups, especially in the MWA and MWA combined with BAI chemotherapy groups, with increased mortality in each group 6 months after the operation.

In the survival analysis, the median survival time of patients treated with MWA+^125^I or MWA+BAI chemotherapy was significantly higher than that of patients treated with MWA alone (*p* < 0.05). This illustrates that MWA combined with local radiotherapy or chemotherapy for lung malignancy can improve the survival time of patients. In a study on tumor-feeding arterial infusion chemotherapy combined with RFA for advanced NSCLC, the 1-, 2-, and 3-year survival rates of patients in the combination group were significantly higher than those of the ablation and perfusion groups [46]. Among the cases of recurrence after liver cancer resection or lung metastasis ablation from liver cancer, CT-guided ^125^I particle-implanted brachytherapy was used as a complementary treatment modality, and significantly prolonged progression-free survival time and overall survival have been observed [47]. RFA combined with ^125^I seed implantation and vertebroplasty exhibited better results for tumors at specific sites, such as spinal metastases [48]. MWA combined with local radiotherapy or chemotherapy in the treatment of tumors not only makes up for the deficiencies of treatment with MWA alone, but also relieves the symptoms caused by the tumor and prolongs the survival time of the patients.

In this study, Cox regression analysis of risk factors affecting the survival of patients with advanced lung cancer and metastatic lung malignancies was performed, including univariate analysis and Cox multivariate analysis, and showed treatment modality as a significant independent influencing factor and protective factor (*p* < 0.05); this also illustrated MWA combined with local radiotherapy or chemotherapy as beneficial for prolonging the survival time. No other independent factors were identified. Studies have shown that MWA, ^125^I seed implantation, BAI chemotherapy, or combined therapy have certain effects on lung primary malignant tumors and lung metastatic tumors. Giulia Lassandro, in a study on lung metastases from liver cancer, noted percutaneous CT-guided thermal ablation in patients with lung metastases from hepatocellular carcinoma as an effective and safe alternative treatment modality for patients who could not undergo surgery [49]. BAI chemotherapy could control the tumor and improve the quality of life in cases of lung metastasis of rectal cancer [50]. In this study, preoperative ZPS was not an independent factor affecting survival time because the interventional department of our hospital strictly regarded ZPS as an important indicator of preoperative evaluation, and patients with scores higher than 2 were not suitable for tumor interventional therapy, so preoperative ZPS was concentrated in the range of 0–2 points. Tumor location was not an independent factor in this regression model because MWA combined with local radiotherapy and chemotherapy had been selected strictly according to the particularity of tumor location, thus improving the treatment effect and prolonging the survival time.

## CONCLUSION

In summary, this retrospective study has found MWA to be a safe and effective treatment modality for advanced lung cancer or metastatic lung malignancies. For patients with specific tumor locations, large tumor diameters, and incomplete ablation by MWA, the treatment can be combined with ^125^I particle implantation or BAI chemotherapy, which may not only control tumor progression, but also improve physical function and prolong patient survival time.

## Figures and Tables

**Fig. (3):**
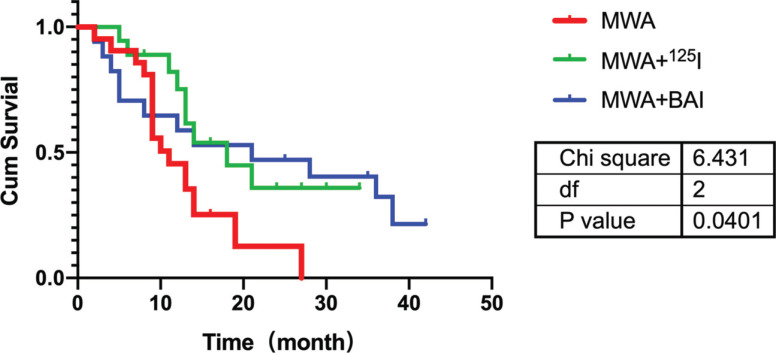
Survival curves of patients in three groups.

**Fig. (5):**
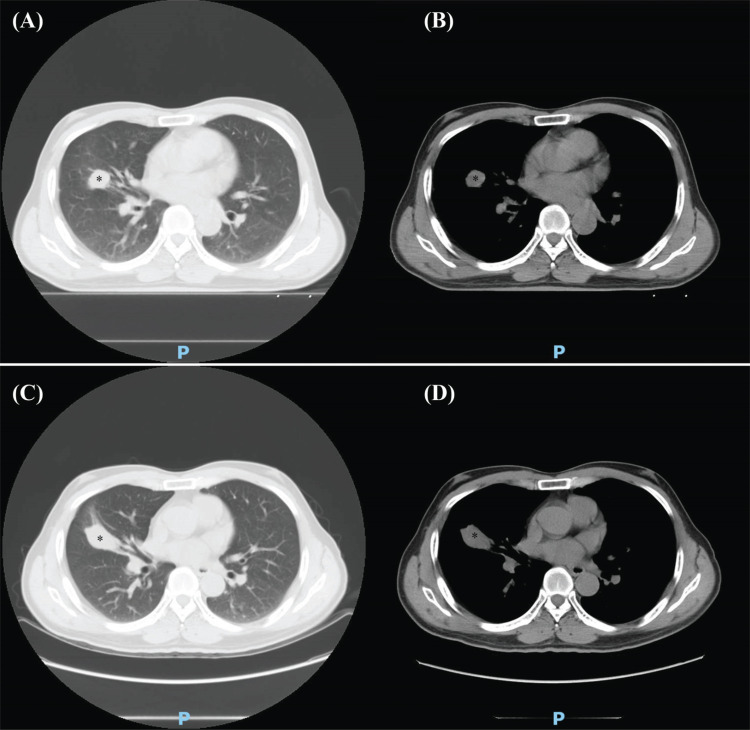
CT results of typical patients in the MWA group before and after the treatment. (**A**) Chest CT lung window of typical patients in the MWA group before the treatment. (**B**) Chest CT mediastinal window of typical patients in the MWA group before the treatment. (**C**) Chest CT lung window of typical patients in the MWA group after the treatment. Chest CT examination 3 months after the operation showed that the right middle lung lesion did not shrink significantly. (**D**) Chest CT mediastinal window of typical patients in the MWA group after the treatment. Chest CT examination 3 months after the operation showed that the right middle lung lesion did not shrink significantly.

**Fig. (6):**
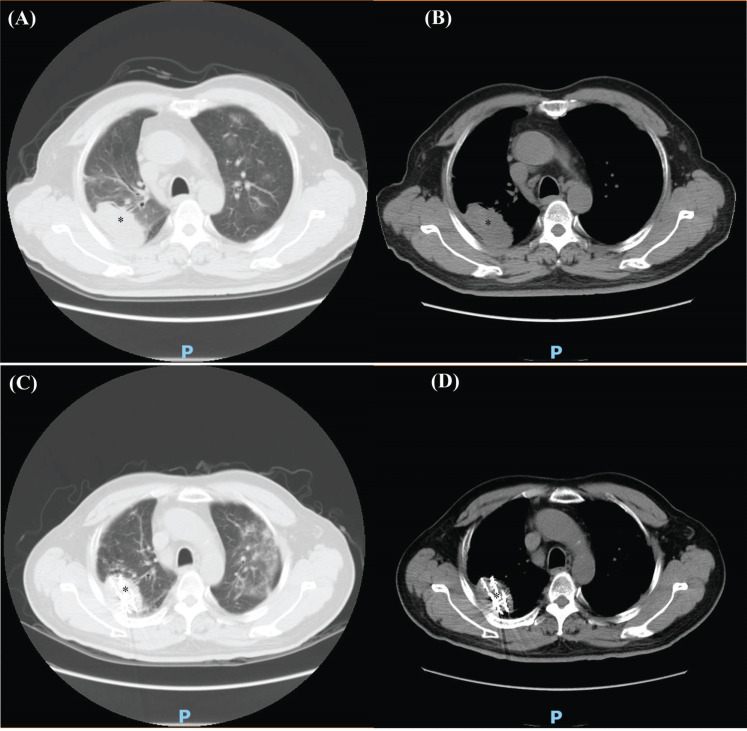
CT results of typical patients in the MWA+^125^I group before and after the treatment. (**A**) Chest CT lung window of typical patients in the MWA +^125^I group before the treatment. (**B**) Chest CT mediastinal window of typical patients in the MWA+^125^I group before the treatment. (**C**) Chest CT lung window of typical patients in the MWA+^125^I group after the treatment. Three months later, lung CT reexamination revealed slight shrinkage of the right upper lung cancer lesion. (**D**) Chest CT mediastinal window of typical patients in the MWA+^125^I group after the treatment. Three months later, lung CT re-examination revealed slight shrinkage of the right upper lung cancer lesion.

**Fig. (7):**
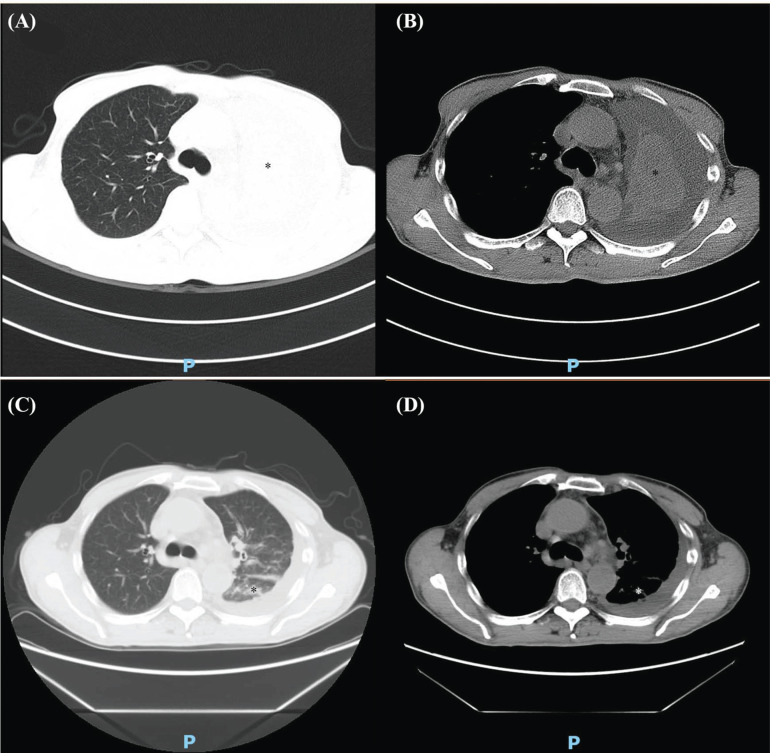
CT results of typical patients in the MWA+BAI group before and after the treatment. (**A**) Chest CT lung window of typical patients in the MWA+BAI group before treatment. (**B**) Chest CT mediastinal window of typical patients in the MWA+BAI group before the treatment. (**C**) Chest CT lung window of typical patients in the MWA+BAI group after the treatment. Three months after the operation, the pulmonary CT showed that the consolidation shadow of the left lung basically disappeared, and scattered nodules and strip shadows could be seen. (**D**) Chest CT mediastinal window of typical patients in the MWA+BAI group after the treatment. Three months after the operation, the pulmonary CT showed that the consolidation shadow of the left lung basically disappeared, and scattered nodules and strip shadows could be seen.

**Table 1:** Basic clinical data of the three groups.

**-**	**MWA(n=21)**	**MWA+^125^I (n=18)**	**MWA+BAI (n=17)**	**F/χ^2^**	***p***
**Gender/n (%)**					
Male	17(80.95)	13(72.22)	13(76.47)	*	0.922
Female	4(19.05)	5(27.78)	4(23.53)		
Age (years)	62.76±11.39	58.39±9.03	58.88±10.67	1.034	0.363
Weight (kg)	60.10±8.50	59.50±8.75	55.74±9.28	1.292	0.283
Height (cm)	167.00±6.08	164.50±7.26	164.71±5.82	0.924	0.403
BMI (kg/m^2^)	21.49±2.37	22.00±3.08	20.55±3.35	1.105	0.339
BSA (m^2^)	1.75±0.13	1.73±0.13	1.69±0.13	1.266	0.29
**Smoking**					
Yes/n (%)	16(76.19)	11(61.11)	11(64.71)	0.571	0.593
No/n (%)	5(23.81)	7(38.89)	6(35.29)		
Hospitalization days	10.00±8.12	12.16±7.38	13.12±6.27	1.098	0.341
ZPS	1.43±0.68	1.56±0.62	1.41±0.51	0.508	0.604
**Lung Cancer Types/n (%)**					
Metastatic carcinoma	7(33.3)	11(61.11)	5(29.42)	4.461	0.107
Primary lung cancer	14(66.67)	7(38.89)	12(70.58)		
**Multiple Lesions/n (%)**					
Yes/n (%)	8(38.10)	11(61.11)	7(41.18)	0.311	0.332
No/n (%)	13(61.90)	7(38.89)	10(58.82)		
**Tumor Location**					
Outer1/3 zone [n (%)]	13(61.90)	4(22.22)	4(23.53)	8.545	0.014
Inner 2/3 zone [n (%)]	8(38.10)	14(78.78)	13(76.47)		
Maximum diameter of tumor(mm)	38.67±23.01	47.61.53±31.26	41.00±23.72	0.595	0.555
Microwave ablation parameters(W)	55.95±9.17	54.72±5.81	53.82±2.81	0.484	0.619
Microwave ablation time(min)	3.55±2.29	3.19±1.51	3.29±2.57	0.139	0.871

**Abbreviations:** BMI, Body Mass Index; BSA, Body Surface Area; ZPS, Zubrod-ECOG-WHO; **Note:** *Fisher's exact method.

**Table 2:** Comparison of short-term efficacy among the three groups.

**Group**	**CR (n)**	**PR (n)**	**SD (n)**	**PD (n)**	**Mean Rank**	**χ^2^/Z**	***p***
MWA	1	1	7	12	19.81	10.422	0.005
MWA+^125^I	2	7	6	3	33.97		
MWA+BAI	4	4	5	4	33.44		
MWA	1	1	7	12	15.26	-2.959	0.004*
MWA+^125^I	2	7	6	3	25.53		
MWA	1	1	7	12	15.55	-2.583	0.014*
MWA+BAI	4	4	5	4	24.38		
MWA+^125^I	2	7	6	3	17.94	-0.034	0.987
MWA+BAI	4	4	5	4	18.06		

**Note:** *α´=0.0167

**Table 3:** Comparison of RR and DCR among the three groups.

**Group**	**Response (n)**	**Non-response (n)**	**RR (%)**	***p***	**Controlled (n)**	**Uncontrolled (n)**	**DCR (%)**	***p***
MWA	2	19	9.52	0.007*	9	12	42.86	0.011*
MWA+^125^I	9	9	50.00		15	3	83.33	
MWA	2	19	9.52	0.012*	9	12	42.86	0.038
MWA+BAI	8	9	47.06		13	4	76.47	
MWA+^125^I	9	9	50.00	0.565	15	3	83.33	0.466
MWA+BAI	8	9	47.06		13	4	76.47	

**Note:** α´=0.0167. * The difference was statistically significant.

**Table 4:** The incidence of complications in the three groups.

	**MWA/n (%)**	**MWA+^125^I/n (%)**	**MWA+BAI/n (%)**	**χ^2^**	***p***
Pneumothorax	2(9.52)	3(16.67)	2(11.76)	*	0.881
Chest pain	3(14.29)	7(38.89)	4(23.53)	*	0.217
Hydrothorax	3(14.29)	1(5.56)	1(5.88)	*	0.608
Pneumonia	2(9.52)	3(16.67)	1(5.88)	*	0.659
Hemoptysis	4(19.05)	4(22.22)	1(5.88)	*	0.428
Myelosuppression	1(4.55)	1(5.88)	2(11.76)	*	0.818
Renal impairment	0	1(5.88)	2(11.76)	*	0.271
Multiple complications	4(19.05)	7(38.89)	4(23.53)	*	0.412
Total	10(47.62)	10(55.56)	9(52.94)	0.258	0.879

**Note:** *Fisher's exact method.

**Table 5:** ZPS of patients in the three groups.

	**MWA**	**MWA+^125^I**	**MWA+BAI**
Preop	1.43±0.68	1.56±0.62	1.41±0.51
1 month	1.43±0.68	1.50±0.51	1.53±0.51
3 months	1.48±0.98	0.50±0.79*#&	0.76±1.39*#&
6 months	1.90±1.33*#	1.22±1.99*#△	1.88±2.39*#△
F time	6.331	Ptime	0.007
F group	0.95	Pgroup	0.393
F time×group	1.911	Ptime×group	0.139

**Note:** Preop: pre-operatively; 1 month: 1 month post-operatively; 3 months: 3 months post-operatively; 6 months: 6 months post-operatively. *Compared to the same group before the operation, *p* < 0.05; # Compared to the same group one month after the operation, *p* < 0.05; △ Compared to 3 months after operation in the same group, *p* < 0.05; & Compared to MWA group at the same time point, *p* < 0.05.

**Table 6:** Univariate Cox regression and multivariate Cox regression.

	**Univariate Cox Regression**		**Multivariate Cox Regression**	
	**HR (95%CI)**	***p***	**HR (95% CI)**	***p***
Age	1.031 (1.000~1.064)	0.052	-	0.118
Gender	0.890 (0.421~1.882)	0.761	-	0.489
Smoking	1.213 (0.603~2.442)	0.589	-	0.997
Lung cancer types	0.718 (0.372~1.387)	0.324	-	0.483
Multiple lesions	0.856 (0.455~1.609)	0.629	-	0.989
Maximum diameter of the tumor	1.005 (0.991~1.018)	0.511	-	0.538
Tumor location	0.886 (0.461~1.705)	0.718	-	0.686
Treatment	0.634(0.413~0.972)	0.036	0.433(0.191~0.984)	0.046
ZPS	0.999 (0.569~1.753)	0.997	-	0.922

## Data Availability

For reasons of confidentiality, the corresponding authors [B.Y. and J.L.] can be contacted for data related to the paper.

## LIST OF ABBREVIATIONS

BAI: Bronchial Arterial Infusion
CT: Computed Tomography
ECG: Electrocardiogram
ECOG: Eastern Cooperative Oncology Group
MWA: Microwave Ablation
NSCLC: Non-Small Cell Lung Cancer
PS: Performance Status
SBRT: Stereotactic Body Radiation Therapy
VATS: Video-Assisted Thoracoscopic Surgery

## STANDARDS OF REPORTING

STROBE guidelines were followed.
